# Die Corona-Pandemie und Multiple Sklerose: Impfungen und deren Implikationen für Patienten – Teil 1: Empfehlungen

**DOI:** 10.1007/s00115-021-01155-4

**Published:** 2021-07-07

**Authors:** Tobias Monschein, Tobias Zrzavy, Micha Löbermann, Alexander Winkelmann, Thomas Berger, Paulus Rommer, Hans-Peter Hartung, Uwe K. Zettl

**Affiliations:** 1 Universitätsklinik für Neurologie, Medizinische Universität Wien, Waehringer Guertel 18–20, 1090 Wien, Österreich; 2 Abteilung für Tropenmedizin und Infektionskrankheiten, Universitätsmedizin Rostock, Rostock, Deutschland; 3 Klinik und Poliklinik für Neurologie, Universitätsmedizin Rostock, Rostock, Deutschland; 4 Klinik für Neurologie, Universitätsklinikum Düsseldorf, Medizinische Fakultät, Heinrich-Heine-Universität, Moorenstraße 5, 40225 Düsseldorf, Deutschland; 5 Klinik und Poliklinik für Neurologie, Neuroimmunologische Sektion, Universitätsmedizin Rostock, Rostock, Deutschland

**Keywords:** Multiple Sklerose, Impfen, SARS-CoV‑2, COVID-19, Pandemie, Multiple sclerosis, Vaccination, SARS-CoV‑2, COVID-19, Pandemic

## Abstract

Die aktuelle COVID-19-Pandemie stellt eine weltweite Gesundheitskrise dar. Nicht zuletzt mit dem Aufkommen diverser Virusmutationen entstehen insbesondere für das Gesundheitswesen laufend neue Herausforderungen. Angesichts der Vielfalt an immunmodulatorischen und immunsuppressiven Therapien für die Multiple Sklerose (MS) sowie der enormen Entwicklungen im Bereich der Vakzinenherstellung besteht ein hoher Informationsbedarf für Menschen mit MS. Ziel dieser Arbeit ist es daher, einen Überblick über MS und COVID-19 sowie die damit einhergehenden Implikationen für MS-Patienten, insbesondere das Impfwesen betreffend, aufzuarbeiten und entsprechende Empfehlungen auszusprechen.

## Abstract (en)

The ongoing COVID-19 pandemic is a global health crisis. New challenges are constantly emerging especially for the healthcare system, not least with the emergence of various viral mutations. Given the variety of immunomodulatory and immunosuppressive therapies for multiple sclerosis (MS) and the immense developments in vaccine production, there is a high need of information for people with MS. The aim of this article is therefore to provide an overview of MS and COVID-19 as well as to clarify the implications for patients with MS, especially regarding vaccination and to formulate appropriate recommendations.

## Einführung

Multiple Sklerose (MS) stellt die häufigste neurologische Erkrankung dar, die zu einer Behinderung im jungen Erwachsenenalter führt und betrifft mehr als 2,3 Mio. Menschen weltweit [9]. Infektionen bergen generell ein Risiko für MS-Patienten, da sie nicht nur einen Pseudoschub auslösen können, sondern auch mit Krankheitsprogression und reduzierter Lebenserwartung assoziiert sind [40, 46, 82]. Dieses infektionsassoziierte Risiko kann sich durch entsprechende Begleiterkrankungen und krankheitsmodifizierende Therapien (DMTs) nochmals weiter erhöhen [23, 39, 54, 62, 81]. Darüber hinaus existieren verschiedene Theorien zur Beteiligung von Infektionen an der Immunpathogenese der MS. Diese umfassen u. a. folgende Mechanismen: „molecular mimicry“, „bystander activation“ und „epitope spreading“ [44].

Mit dem Ausbruch der COVID-19-Pandemie Ende 2019 in der Provinz Hubei in China stehen daher insbesondere Menschen mit MS vor neuen Herausforderungen. Hier kommen nicht nur im Bereich der Patientenversorgung und Immuntherapien Fragen auf, sondern vor allem auch Fragen, die das Impfwesen betreffen [29, 57, 69]. Inzwischen wurden zahlreiche Impfstoffe weltweit zugelassen, darunter 2 mRNA- und 2 Vektorimpfstoffe in der Europäischen Union (EU; [4, 59, 77, 85]). Angesichts der enormen Fortschritte im Bereich der Vakzinenherstellung auf der einen Seite und diverser Virusmutationen auf der anderen Seite ist ein hoher Informationsbedarf, sowohl für das Gesundheitspersonal als auch Menschen mit MS, gegeben. Ziel dieser Arbeit ist es daher, einen Überblick über COVID-19, Manifestationen als auch das Impfwesen und dessen technologische Fortschritte zu geben und deren Implikationen für Menschen mit MS herauszuarbeiten und entsprechende Empfehlungen zu generieren.

## COVID-19 Hintergrund

COVID 19 ist eine Infektionskrankheit, die durch das hochansteckende RNA-Virus SARS-CoV‑2 („severe-acute-respiratory-syndrome corona-virus-2“) verursacht wird. Insgesamt wurden bisher 7 humanpathogene Coronaviren beschrieben, darunter auch das „severe acute respiratory syndrome corona-virus“ (SARS-CoV, Ausbruch 2002) sowie das „middle east respiratory syndrome coronavirus“ (MERS-CoV, Ausbruch 2012) mit ebenfalls zoonotischen Ursprüngen [13]. Des Weiteren konnte gezeigt werden, dass eine hohe genetische Übereinstimmung (bis zu 79 %) mit anderen Coronaviren gegeben ist [36]. Die Übertragung auf den Menschen ist bislang nicht geklärt, es gibt jedoch Evidenz aus phylogenetischen Studien, dass diese am ehesten von der Fledermaus und dem Pangolin ausgeht [19]. Bis zum 24.04.2021 wurden von Seiten der WHO 145.216.414 bestätigte SARS-CoV-2-Fälle sowie 3.079.390 Todesfälle registriert [86]. Die aktuelle COVID-19-Pandemie stellt damit eine weltweite Gesundheitskrise dar. Eines der Hauptprobleme von COVID-19 ist die Übertragung von SARS-CoV‑2 auch von asymptomatischen und präsymptomatischen Personen, im Gegensatz zu SARS-CoV [78, 79]. Der hauptsächliche Infektionsweg scheint via Aerosole/Tröpfcheninfektion zu erfolgen, es werden jedoch auch andere Infektionswege wie via Kontakt oder kontaminierte Oberflächen diskutiert [87].

Des Weiteren bindet SARS-CoV‑2, ähnlich wie SARS-CoV, an „angiotensin-converting enzyme 2“ (ACE-2), um in respiratorische Epithelzellen einzudringen, weist jedoch eine 10- bis 20-fach höhere Bindungsaffinität als SARS-CoV auf [33]. Nach dem Eindringen in die respiratorischen Epithelzellen beginnt SARS-CoV‑2 mit der Replikation und raschen Ausbreitung, bis in die alveolären Epithelzellen der Lunge. Das angeborene Immunsystem bewerkstelligt eine erste unspezifische, sofortige Immunreaktion. Dabei wird das Spike-Protein von Erregererkennungsrezeptoren („pathogen-associated molecular patterns“, PAMPS) erkannt und führt über diverse Signalkaskaden zu einer Aktivierung von Immunzellen mit konsekutiver Freisetzung von Signalmolekülen, die wiederum eine Rekrutierung weiterer Immunzellen begünstigen z. B. durch eine gesteigerte Interferonproduktion. Dies wiederum verzögert die Virusausbreitung und führt in der Folge zur Aktivierung des adaptiven Immunsystems. Durch Präsentation spezifischer Abschnitte des Virus mittels Haupthistokompatibilitätskomplex I oder II antigenpräsentierender Zellen, wie z. B. Makrophagen, folgt die Einleitung einer virusspezifischen Immunantwort mit Aktivierung und klonaler Expansion von CD4^+^- sowie CD8^+^- T-Zellen und die Generierung von Plasmazellen mit konsekutiver Produktion von Antikörpern [63]. Durch die T‑Zell-Aktivierung, insbesondere CD8^+^- T-Zellen, kommt es zu einer Elimination von bereits infizierten Zellen, während die CD4^+^-T-Zellen hier unterstützend durch das Instruieren von B‑Zellen, das Unterstützen von CD8^+^-T-Zellen, das Rekrutieren angeborener Immunzellen sowie eine direkte antivirale Aktivität wirken [63, 67, 70, 74]. Je nach Schwere der Infektion kann eine rapide Erregerausbreitung zusammen mit begleitender Gewebedestruktion ein Zytokin-Sturm-Syndrom (CSS) verursachen [16, 42]. Dieses kann in einem akuten Atemnotsyndrom (ARDS) resultieren, wofür wiederum Alter und Komorbiditäten die wichtigsten Risikofaktoren darstellen [58, 84].

Insbesondere für Patienten mit Autoimmunerkrankungen wie der MS und gegebenenfalls entsprechender immunmodulatorischer und/oder immunsuppressiver Therapie ist es von Interesse, eine entsprechende Immunantwort nach durchgemachter SARS-CoV-2-Infektion und/oder Impfung nachzuweisen (unter der Voraussetzung verfügbarer Cut-offs für eine Immunprotektion). Für den Nachweis der B‑Zell-Immunität steht beispielsweise der ELISA („enzyme-linked-immunoassay“) zur Detektion des Antikörpertiters zur Verfügung. Mittels zellbasierter Verfahren (Neutralisationstest) kann darüber hinaus der Anteil an neutralisierenden Antikörpern nachgewiesen werden. Ein Teil der Antiköper wirkt neutralisierend, andere erfüllen Funktionen wie Opsonierung oder Komplementaktivierung [17, 65]. Für den Nachweis einer T‑Zell-Immunität können Verfahren wie der ELISpot („enzyme linked immuno spot assay“) oder eine Durchflusszytometrie (z. B. „fluorescence-activated cell sorting“ [FACS ]) angewendet werden [53]. In einer rezenten Studie von Achiron et al. wurde die humorale Immunantwort anhand des Antikörpertiters (ELISA) bei Gesunden, MS-Patienten mit und ohne Therapie verglichen und es konnte eine adäquate Immunantwort für alle, ausgenommen einem Teil der Patienten mit Ocrelizumab sowie den meisten Patienten mit Fingolimod als DMT, gezeigt werden [2].

## Neurologische Beteiligungen bei COVID-19

Die Manifestationen von COVID-19 gestalten sich vielseitig. Die Symptome reichen von Fieber, trockenem Husten und Müdigkeit bis hin zu respiratorischem Versagen mit fatalem Ausgang [24, 73, 80]. Außerdem konnte gezeigt werden, dass ACE‑2 nicht nur in der Lunge, sondern auch im Ösophagus, Ileum und Kolon exprimiert wird. Angesichts dessen sowie Durchfallraten von bis zu 50 % stellt der Gastrointestinaltrakt ebenfalls eine mögliche Eintrittspforte für das Virus dar [14, 83]. Neben diesen systemischen Manifestationen gibt es zunehmend Evidenz für eine Mitbeteiligung des Nervensystems. Dies kann von einer milden Hyposmie über parainfektiöse Syndrome bis hin zu Enzephalopathien und Schlaganfällen gehen [18, 56].

Neurologische Mitbeteiligungen finden sich darüber hinaus auch häufig bei hospitalisierten Kindern und Jugendlichen [31]. Eine mögliche Route ist die Migration in das ZNS mittels axonalen Transports über olfaktorische Neurone. Auffällig sind in diesem Zusammenhang auch die häufigen Hyposmien sowie MRT-Veränderungen im olfaktorischen Kortex [60]. Eine weitere Möglichkeit wäre das Überwinden der Blut-Hirn-Schranke (BHS), was via Internalisierung und/oder inflammationsgetriggerte Funktionsstörung der BHS erfolgen kann [25]. Eine dritte Möglichkeit stellen infizierte Immunzellen dar, die ins Gehirn einwandern und auch als Reservoir dienen können [6].

Überblicksmäßig ergeben sich 5 Hauptkategorien für neurologische Manifestationen im Zuge von COVID-19: (1) Enzephalopathie, (2) entzündliche ZNS-Syndrome wie Enzephalitis oder ADEM, (3) ischämische Schlaganfälle, (4) periphere Beteiligungen wie das Guillain-Barre-Syndrom (GBS) und (5) andere Manifestationen [56]. Bezüglich GBS und COVID-19 gibt es widersprüchliche Befunde. So zeigen einzelne epidemiologische Untersuchungen keine Assoziation von GBS mit COVID-19 [28]. Darüber hinaus wurde in einer rezenten Arbeit von Nalbandian et al. das postakute COVID-19-Syndrom (> 4 Wochen nach Infektion) beschrieben, welches sich in bis zu 38 % der Fälle in Form neurologischer Symptome äußert (Fatigue, Kopfschmerzen, Dysautonomie, kognitive Beeinträchtigung; [49]). Einen tabellarischen Überblick mit den entsprechenden einzelnen Diagnosen sowie vermuteten Pathomechanismen soll Tab. 1 geben.

**Table 1:** 

Neurologische Manifestation (COVID-19)	Beschriebene Krankheitsbilder	Pathomechanismus
Enzephalopathie	Delir, Psychose, Vigilanzminderung	Multifaktoriell (Sepsis, Hypoxie, Immunhyperstimulation/CSS)
Entzündliche ZNS-Syndrome	Enzephalitis, ADEM (teils mit ICB, teils mit Nekrose), transverse Myelitis	Para-/postinfektiöser Mechanismus (z. B.: „molecular mimicry“, „bystander activation“, „epitope spreading“)
Schlaganfall	Ischämisch, Mikrohämorrhagien	Begleitvaskulitis, prothrombotischer Status/Hyperkoagulabilität, endotheliale Dysfunktion für Mikrohämorrhagien
Periphere Beteiligung	GBS, Plexopathie	Para-/postinfektiöser Mechanismus (z. B.: „molecular mimicry“, „bystander activation“, „epitope spreading“)
Postakutes COVID-19-Syndrom (38 % neurologische Manifestationen)	Fatigue, Kopfschmerzen, Dysautonomie, kognitive Beeinträchtigung	Immundysregulation, Inflammation, mikrovaskuläre Thrombosen, iatrogene Wirkungen von Medikamenten
Andere	Hyposmie/Anosmie, Myelopathie (MR-negativ), bilaterale Abduzensparese, nonkonvulsiver Status epilepticus (NCSE), bakterieller Abszess	Migration in das ZNS via N. olfactorius, bilaterale Abduzensparese bei idiopathischer intrakranieller Hypertension, NCSE bei pädiatrischen Patienten mit bekannter Epilepsie

## Multiple Sklerose und die COVID-19-Pandemie

Multiple-Sklerose-Patienten scheinen per se nicht einem erhöhten Risiko für eine SARS-CoV-2-Infektion oder einen schwereren Verlauf zu unterliegen [26, 55]. Daten aus Italien und Frankreich konnten, neben bekannten allgemeinen kardiovaskulären Risikofaktoren wie Adipositas, vor allem Alter und Behinderungsgrad (Expanded Disability Status Scale, EDSS) als Risikofaktoren für einen schwereren Verlauf von COVID-19 bei MS-Patienten herausarbeiten [35, 72]. Des Weiteren wurde in der italienischen Studie eine erhöhte Anzahl an schwereren COVID-19-Fällen unter MS-Patienten mit B‑Zell-depletierenden Therapien detektiert. Im Gegensatz dazu konnte in der französischen Studie keine Assoziation zwischen DMTs und COVID-19-Schwere aufgezeigt werden. Mögliche Risiken, die im Zusammenhang mit B‑Zell-depletierenden Therapien diskutiert werden, sind eine beeinträchtigte antivirale erworbene Immunität (reduzierte Antikörpertiter sowie reduzierte T‑ und B‑Zell-Interaktion), ein erhöhtes Reinfektionsrisiko, eine beeinträchtigte Impfantwort sowie die assoziierte mögliche Hypogammaglobulinämie.

Auf der anderen Seite gibt es diverse Komplikationen einer SARS-CoV-2-Infektion, z. B. Hyperinflammation/CSS und dadurch in weiterer Folge das für einen fatalen Verlauf entscheidende ARDS, die durch eine solche Therapie positiv beeinflusst werden könnten [43].

In diesem Zusammenhang sind mögliche positive Einflüsse auf die COVID-19-Schwere auch durch Interferon‑β (antivirale Wirkung) oder monoklonale Antikörper gegen den Interleukin-6-Rezeptor (Tocilizumab) beschrieben worden [21, 27, 64]. Ergänzend dazu zeigte eine Studie von Alonso et al. eine erhöhte Hospitalisierungsrate bei NMOSD-Patienten (*n* = 16) gegenüber MS-Patienten (*n* = 129). Des Weiteren starb in dieser Studie ein Drittel der NMOSD-Patienten, von denen alle unter einer Therapie mit Rituximab standen. Hier gilt es jedoch, die geringe Gesamtfallzahl an NMOSD-Patienten (*n* = 16) zu berücksichtigen [3].

Unter Zusammenschau rezenter Evidenz bleibt jedoch weiterhin ungeklärt, inwiefern eine COVID-19-Infektion und deren Verlauf durch DMTs beeinflusst werden [30]. Angesichts der fortbestehenden COVID-19-Pandemie werden zukünftige umfangreichere Therapieregisterdaten von MS-Patienten mehr Klarheit über den Einfluss von DMTs bringen. Insgesamt scheinen jedoch Risikofaktoren wie Alter, Behinderungsgrad (EDSS) und Komorbiditäten für den Verlauf bzw. die Schwere einer SARS-CoV-2-Infektion viel entscheidender zu sein, als DMTs [7, 10, 23, 38, 39, 41, 62]. Auf dem bisherigen Wissensstand aufbauend wird gemäß den jeweiligen regionalen Empfehlungen einheitlich die Fortführung immunmodulatorischer Therapien wie bisher empfohlen. Bei zyklischen Therapien wie Ocrelizumab, Rituximab, Alemtuzumab oder Cladribin kann jedoch je nach individueller Situation (Komorbiditäten, Alter, EDSS, Lymphozytenzahl, Krankheitsaktivität) und in Absprache mit dem behandelnden Neurologen eine Verschiebung des nächsten Therapiezyklus sinnvoll sein [5, 8, 37, 88–91].

## Impfempfehlungen – COVID-19 und MS

Insgesamt wurden bisher 14 neue Impfstoffe zugelassen, die sich gegen SARS-CoV‑2 richten [4, 11, 34, 50, 51, 59, 77, 85, 92]. Innerhalb der EU wurden bereits 4 Impfstoffe (2 mRNA- und 2 nichtreplizierende Vektorvirusimpfstoffe) zugelassen. Für all diese 4 Impfstoffe liegen gute Wirksamkeitsdaten (67–95 %) vor [4, 59, 77, 85]. Darüber hinaus konnte in einer schottischen postapprovalen Studie eine 94 %ige Reduktion der Krankenhausaufenthalte gezeigt werden, bei einem Studienkollektiv von mehrheitlich über 80-Jährigen [93]. Eine Folgestudie aus Schottland ergab für den BionTech/Pfizer- und AstraZeneca-Impfstoff bereits 28 bis 34 Tage nach der ersten Impfung eine Reduktion der COVID-19-bedingten Spitalsaufenthalte um 88–91 % [75]. Ähnliche Ergebnisse liefert eine Studie an Mitarbeitern im Gesundheitswesen [22]. Dem folgend gibt es eine klare Empfehlung der WHO für die Anwendung bei > 65-jährigen Personen, insbesondere den AstraZeneca-Impfstoff betreffend (in der Zulassungsstudie nur 9,8 % > 65 Jahre) [77, 94]. Auch das Sicherheitsprofil der in der EU zugelassenen Impfstoffe ist gut, insbesondere unter Berücksichtigung von bereits beinahe 900 Mio. verabreichten Impfstoffdosen weltweit (laut WHO bis zum 22.04.2021; [1, 86]).

In Zusammenhang damit muss jedoch angemerkt werden, dass bis zum 04.04.2021 insgesamt 169 Fälle von zerebraler Venenthrombose (CVT) und 53 Fälle von splanchnischer Venenthrombose an EudraVigilance nach AstraZeneca (®Vaxzevria) gemeldet wurden, bei über 34 Mio. geimpften Menschen in der EU und Großbritannien [12, 48, 66, 68, 95]. Diese Daten ändern nichts an den Empfehlungen des Sicherheitsausschusses (PRAC) der EMA für den Impfstoff AstraZeneca (®Vaxzevria), dass der Nutzen dem Risiko klar überwiegt. So hat beispielsweise die STIKO in Deutschland die Empfehlung herausgegeben, dass Menschen unter 60 Jahren nicht mehr mit diesem Impfstoff geimpft werden sollen und Personen mit einer bereits erhaltenen Impfung als 2. Impfung einen mRNA-Impfstoff im Sinne eines heterologen Impfschemas erhalten sollen [96]. In einer Arbeit von Greinacher et al. wird die Bildung plättchenaktivierender Antikörper als möglicher Mechanismus für eine prothrombotische Thrombozytopenie nach der Impfung postuliert [20]. Auch im Zusammenhang mit dem Impfstoff von Johnson&Johnson wurden 6 CVTs berichtet. Diesbezüglich gab es jedoch eine Sicherheitsüberprüfung durch die FDA (Food and Drug Administration) und das CDC (Centers for Disease Control and Prevention) und es wurde konsekutiv die Empfehlung zur Pausierung der Notzulassung wieder aufgehoben [97]. Nichtsdestotrotz können aktuell potenzielle Langezeitschäden nicht vollends ausgeschlossen werden, weswegen ein entsprechendes Surveillance-Programm notwendig ist. Des Weiteren wurden im Falle des AstraZeneca-Impfstoffs 3 Myelitisfälle berichtet, auch wenn diese von einem unabhängigen Expertenkomitee als unwahrscheinlich mit der Impfung im Zusammenhang stehend beurteilt wurden [77].

Darüber hinaus haben sich durch die rezente Zulassung des Johnson&Johnson-Impfstoffs in der EU (11.03.2021), nach dem dieser bereits Ende Februar eine Notzulassung durch die FDA erhalten hatte, die Alternativen der Impfstoffe erweitert [85, 98]. Der Vorteil dieses Impfstoffs könnte, insbesondere im Kampf gegen die aufkommenden Virusmutationen sein, dass er nur einmalig verabreicht werden muss (Gefahr der Resistenzbildung geringer; [47]). Die Wirksamkeit dieses Impfstoffes lag nach 14 Tagen bei 66 % und nach 28 Tagen zwischen 57–72 % und ist damit am ehesten vergleichbar mit dem AstraZeneca-Impfstoff [99]. Bezüglich des russischen Sputnik-V-Impfstoffs wurden am 02.02.2021 die Phase-III-Studiendaten veröffentlicht, mit einer Wirksamkeit von 91,6 %. Das Sicherheitsprofil dieser beiden nichtreplizierenden Vektorvirusimpfstoffe ist, soweit dies aus den bisherigen Veröffentlichungen ersichtlich ist, ebenfalls gut [34, 85, 99]. Insgesamt sind alle bis dato in der EU zugelassenen SARS-CoV-2-Impfstoffe für MS-Patienten zu empfehlen. Angesichts der 3 Myelitisfälle in den AstraZeneca-Impfstoffstudien sowie der CVT-Fälle als auch der tendenziell höheren Wirksamkeit der mRNA-Impfstoffe, ist hier eine gewisse Überlegenheit der mRNA-Impfstoffe gegeben. Dies entspricht auch den jeweiligen regionalen Empfehlungen [88–91]. Insbesondere den AstraZeneca-Imfpstoff betreffend müssen die Empfehlungen der STIKO berücksichtigt werden (nur bei > 60-Jährigen).

Da es sich bei diesen 4 Impfstoffen um keine Lebendimpfstoffe handelt, besteht keine Gefahr einer Erkrankung, durch die Impfung. Gegebenenfalls kann es bei Patienten unter Immuntherapie zu einer reduzierten Immunantwort kommen [32, 76]. Insbesondere im Falle einer zyklischen Immuntherapie (Ocrelizumab, Rituximab, Alemtuzumab, Cladribin), kann ein entsprechender Aufschub der nächsten Gabe sinnvoll sein, um möglichst eine protektive Immunantwort generieren zu können. Alle Impfungen sollten ausschließlich in Absprache mit dem behandelnden Neurologen und unter Berücksichtigung der einzuhaltenden Zeitintervalle erfolgen.

## Schlussfolgerungen

COVID-19 ist eine Pandemie, die zu einem weltweiten Ausnahmezustand geführt hat, der viele Bereiche trifft, insbesondere das Gesundheitswesen. Seit Anbeginn der Pandemie haben sich beinahe 150 Mio. Menschen mit SARS-CoV‑2 infiziert und sind über 3 Mio. Menschen an COVID-19 verstorben [86]. Innerhalb des letzten Jahres gab es enorme wissenschaftliche Fortschritte, sodass inzwischen weltweit 14 Impfstoffe gegen SARS-CoV‑2 zugelassen werden konnten und beinahe 900 Mio. Impfdosen (laut WHO bis zum 22.04.2021) verabreicht werden konnten [4, 11, 34, 50, 51, 59, 77, 85, 86, 92]. Gerade im Zeitalter der immunmodulatorischen und immunsuppressiven Therapien sowie angesichts der Entwicklungen im Bereich der Impfstofftechnologien stehen Menschen mit Multipler Sklerose vor neuartigen Herausforderungen und Informationsbedarf. In diesem Zusammenhang ist es wichtig, zu betonen, dass Menschen mit MS kein generell erhöhtes Risiko haben, an COVID-19 zu erkranken oder einen schwereren Verlauf zu erleiden [35, 71]. Vielmehr scheinen Komorbiditäten, Alter und Behinderungsgrad (EDSS) entscheidend zu sein [10, 29, 30]. Es wird daher allen MS-Patienten empfohlen, ihre Immuntherapie wie bisher fortzuführen, immer in Absprache mit dem behandelnden Neurologen. Gerade bei zyklischen Therapien (Ocrelizumab, Rituximab, Alemtuzumab, Cladribin) kann eine mögliche Aufschiebung der nächsten Dosis sinnvoll sein [89]. Derzeit wird allen MS-Patienten das Impfen gegen SARS-CoV‑2 entsprechend den regionalen Vorgaben empfohlen. Nur so kann der COVID-19-Pandemie Einhalt geboten werden und ein Ende dieser weltweiten Gesundheitskrise erreicht werden.
